# Phytochemical Screening and Protective Effects of *Hancornia speciosa* Leaf Extract Against Fatty Acid-Induced Lipotoxicity in HepG2 Cells

**DOI:** 10.3390/molecules31203707

**Published:** 2026-10-16

**Authors:** Maria Júlia Maia Gonçalves Granato, Frank Machado Silva, Vinícius Marques Arruda, Adilson Júnior Alves Bezerra, Bruno Quintanilha Faria, Mayara Medeiros de Freitas Carvalho, Terezinha Aparecida Teixeira, Tiara da Costa Silva, Diego Godina Prado, Hebréia Oliveira Almeida Souza, Joyce Ferreira da Costa Guerra

**Affiliations:** 1 Institute of Biotechnology, Federal University of Uberlândia, Uberlândia 38405-319, Brazil; maria.granato@ufu.br (M.J.M.G.G.); frank.machado@ufu.br (F.M.S.); vinicius.arruda@ufu.br (V.M.A.); adilson.bezerra@ufu.br (A.J.A.B.); terezinha.teixeira@ufu.br (T.A.T.); tiara.silva@uftm.edu.br (T.d.C.S.); hebreia@ufu.br (H.O.A.S.); 2 Department of Pharmacy, Federal University of Paraná, Curitiba 80210-170, Brazil; bruno.quintanilha@ufpr.br; 3 Department of Biological Sciences, Federal University of Ouro Preto, Ouro Preto 35400-000, Brazil; mayaracarvalho@professor.multivix.edu.br; 4 Institute of Exact, Natural Sciences and Education, Federal University of Triângulo Mineiro, Uberaba 38064-200, Brazil; 5 Institute of Chemistry, Federal University of Uberlândia, Uberlândia 38400-902, Brazil; diegodina@ufu.br

**Keywords:** antioxidants, steatosis, mangabeira, oxidative stress, phytochemical screening, plant metabolites, polyphenols, untargeted metabolomics

## Abstract

Hepatic steatosis is the hallmark of metabolic dysfunction-associated steatotic liver disease (MASLD), the most prevalent chronic liver disease worldwide. Lipotoxicity, resulting from excessive accumulation of fatty acids and bioactive lipid intermediates, is associated with increased oxidative stress, potentially leading to cell death. Thus, antioxidant bioactive compounds are considered promising therapeutic agents. This study characterized the phytochemical composition and evaluated the effects of an aqueous extract of *Hancornia speciosa* leaves (EH) on HepG2 cells with fatty acid-induced lipid accumulation. HPLC-ESI-MS/MS analysis annotated 17 compounds in EH, predominantly phenolic compounds. Exposure of HepG2 cells to oleic and palmitic acids increased lipid accumulation, cytotoxicity, and antioxidant enzyme activities. Treatment with EH at 50 (EH50) and 100 µg/mL reduced lipid accumulation and reactive oxygen species and restored catalase and glutathione S-transferase activities to control levels. Notably, only EH50 showed reduced cytotoxicity. Untargeted metabolomic profiling showed an increase in intracellular abundance of the feature annotated as C16 sphinganine following fatty acid exposure and lower abundance after EH50 treatment. These findings indicate that *H. speciosa* leaf extract attenuates fatty acid-induced lipid accumulation, lipotoxicity, and redox imbalance in HepG2 cells, and suggest that its protective effects may be associated with sphingolipid-related metabolism, thereby strengthening evidence for the species’ therapeutic potential and supporting further studies to elucidate the underlying mechanisms.

## 1. Introduction

Hepatic steatosis is a key feature of metabolic dysfunction-associated steatotic liver disease (MASLD), currently the most prevalent chronic liver disease worldwide, affecting approximately 38% of the global population and representing one of the leading causes of liver-related morbidity and mortality [1].

Lipid accumulation within hepatocytes initiates progressive metabolic impairment, mainly driven by lipotoxicity, which is characterized by an excess of potentially toxic bioactive lipid intermediates, such as diacylglycerols, free cholesterol, sphingolipids, and ceramides [2,3]. This process increases the production of reactive oxygen species (ROS), leading to oxidative stress, which in turn exacerbates mitochondrial dysfunction and activates pro-inflammatory [4,5] and pro-fibrotic signaling pathways, contributing to hepatocellular damage and disease progression [6,7].

As the accumulation of lipids in hepatocytes is considered a pathological hallmark, in vitro models of steatosis provide valuable tools for elucidating biochemical pathways and screening bioactive compounds. HepG2 cells, a human hepatoblastoma-derived cell line with hepatocyte-like characteristics, are widely used as a cellular model to investigate metabolic alterations associated with hepatic steatosis [8]. Lipid overload is commonly induced by exposing the cells to fatty acids, particularly palmitic and oleic acids, either individually or in combination. Fatty acid exposure promotes intracellular triglyceride accumulation and can induce oxidative stress, mitochondrial dysfunction, and lipotoxicity [9,10,11,12,13], thereby mimicking key features of the disease.

Natural bioactive compounds with antioxidant properties have gained increasing attention as potential therapeutic agents [14,15,16,17]. Plant-derived secondary metabolites, particularly polyphenols like flavonoids, exhibit antioxidant, anti-inflammatory, hypolipidemic, and hepatoprotective effects by modulating several key signaling pathways [17,18,19,20,21,22].

The Brazilian Cerrado, recognized as one of the world’s biodiversity hotspots, represents a rich source of unexplored plant species, underscoring the need for conservation efforts and scientific investigation of its native species as potential sources of secondary metabolites with biotechnological and pharmacological applications [23,24]. Among these species, *Hancornia speciosa* Gomes (Apocynaceae), commonly known as mangabeira, has been traditionally used for medicinal purposes, with preparations typically obtained by infusion or decoction of different plant parts, including leaves, bark, roots, and fruits. Previous studies have reported antioxidant, anti-inflammatory, hypoglycemic, hypolipidemic, and hepatoprotective activities in extracts derived from this species, which have been associated with its phenolic and flavonoid constituents [24,25,26,27,28]. Furthermore, a previous study showed that the fruit pulp of *H. speciosa* exerts hepatoprotective, hypolipidemic, and antioxidant effects in rats fed a high-fat diet [29]. Despite these effects, the biological activities of extracts from *H. speciosa* leaves remain poorly investigated.

Therefore, the present study aimed to characterize the phytochemical composition of an aqueous extract of *H. speciosa* leaves and investigate its effects on lipid content, redox homeostasis, and the untargeted metabolomic profile of HepG2 cells with fatty acid-induced lipid accumulation. Our data showed that *H. speciosa* leaf extract reduces lipid accumulation, improves antioxidant defenses, and attenuates lipotoxicity in lipid-overloaded HepG2 cells. These protective effects were associated with changes in metabolic features putatively annotated as sphingolipid-related metabolites, suggesting that the modulation of sphingolipid metabolism may be involved in the cellular response to EH.

## 2. Results

### 2.1. Phytochemical Characterization of Hancornia speciosa Leaf Aqueous Extract

The phytochemical profiling of the aqueous extract of *H. speciosa* leaves (EH) was comprehensively investigated to identify its bioactive constituents and evaluate its antioxidant capacity. High-performance liquid chromatography coupled with electrospray ionization tandem mass spectrometry (HPLC-ESI-MS/MS) allowed the annotation of bioactive compounds based on retention times, exact precursor ion masses, and their respective MS/MS fragmentation patterns. The annotated compounds and their spectrometric parameters are presented in Table 1.

**Figure 1:**
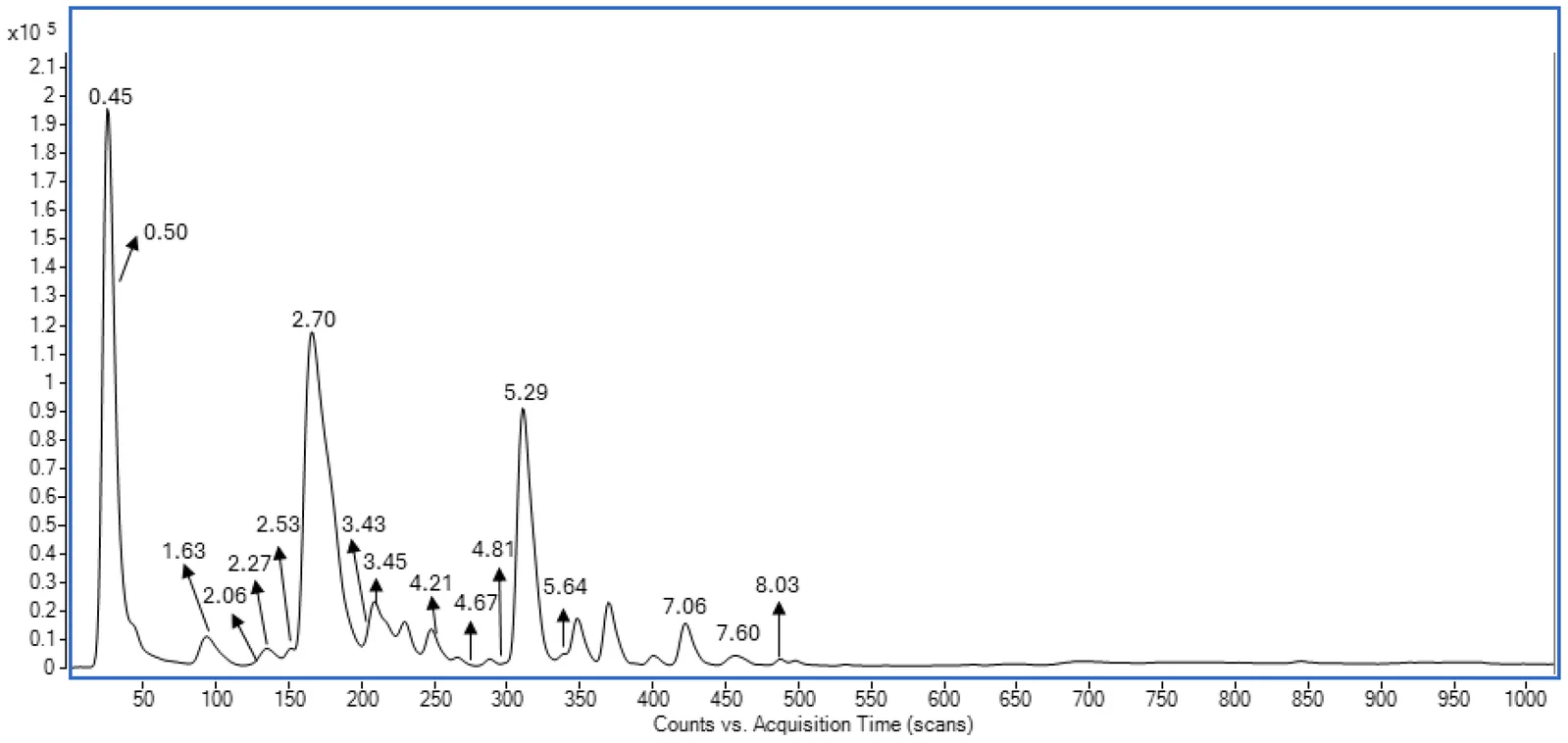
Base peak chromatogram of EH obtained by HPLC-ESI-MS/MS. Compounds were annotated based on retention times, precursor ion exact masses, and MS/MS fragmentation patterns compared with literature data.

The chemical composition analysis of the aqueous extract of *H. speciosa* leaves resulted in the annotation of five phenolic acids, two organic acids, three terpenoids, two oxygenated fatty acids, and five flavonoids. The base peak chromatogram (Figure 1) showed three peaks with higher intensities, corresponding to quinic acid (*m*/*z* = 191.0562; tR = 0.45 min), malic acid (*m*/*z* = 133.0141; tR = 0.50 min), caffeoylquinic acid isomer I (*m*/*z* = 353.0882; tR = 1.63 min), and rutin (*m*/*z* = 609.1463; tR = 5.29 min).

**Figure 2:**
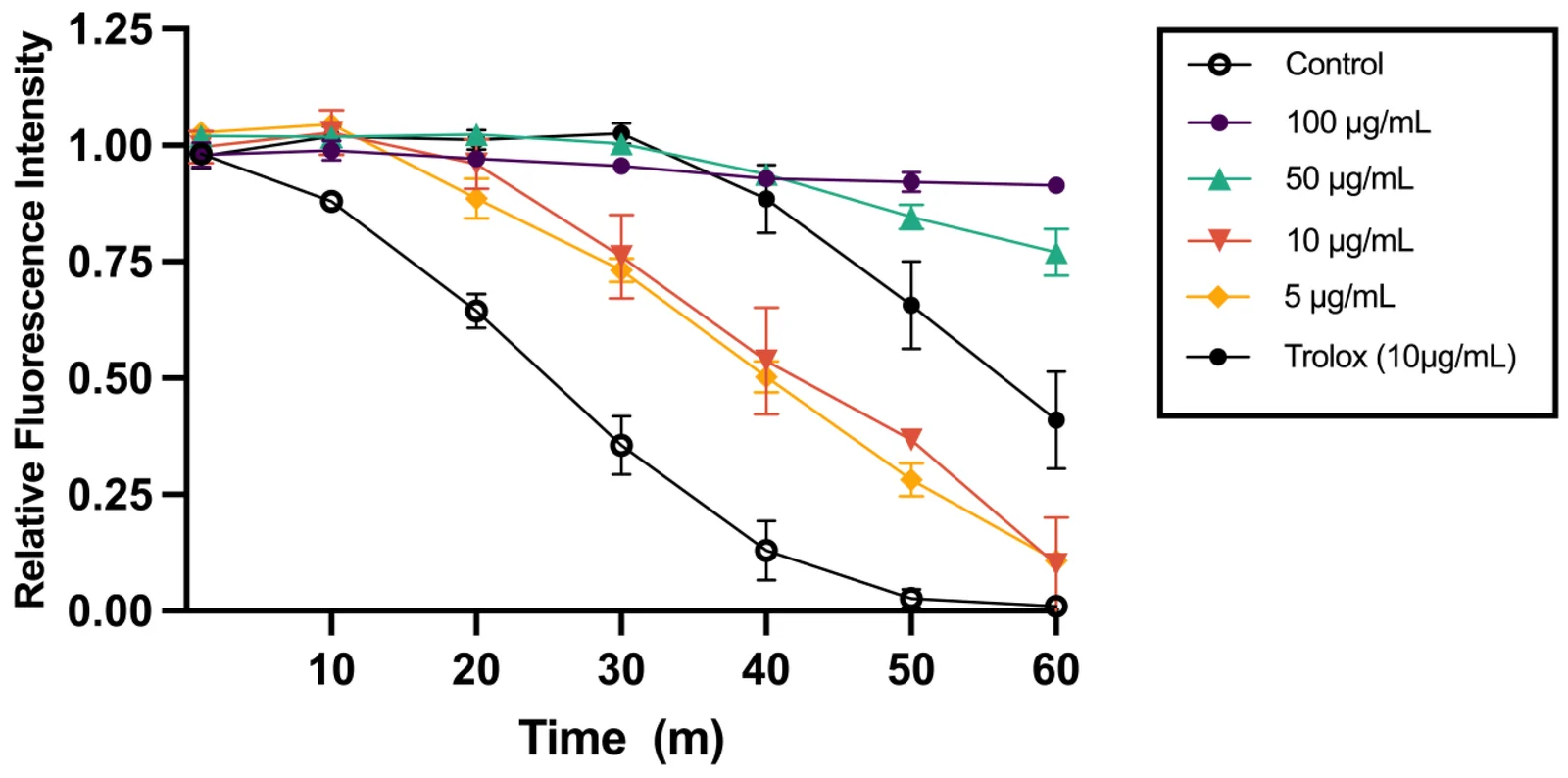
Oxygen radical scavenging capacity of *H. speciosa* leaf aqueous extract (EH). Fluorescence decay kinetics curves obtained over 60 min in the oxygen radical absorbance capacity (ORAC) assay in the presence of increasing concentrations of EH (5–1000 µg/mL) or Trolox (10 µg/mL; 40 µM). Trolox, a water-soluble analog of vitamin E, was used as a reference antioxidant. All samples were analyzed in triplicate, and data are presented as mean ± standard deviation.

In addition to the chromatographic profiling, total polyphenolic content and Oxygen Radical Absorbance Capacity (ORAC) antioxidant capacity of EH were evaluated. The extract presented a high concentration of total phenolics (173.02 ± 2.01 mg gallic acid equivalents (GAE)/g freeze-dried extract) and total flavonoids (55.33 ± 2.63 mg equivalents (QE)/g freeze-dried extract). Consistent with this polyphenol-rich composition, EH demonstrated high antioxidant capacity, with an ORAC value of 423.81 ± 6.66 µmol Trolox equivalents (TE)/g freeze-dried extract. Peroxyl radical scavenging capacity was particularly pronounced at 50 and 100 µg/mL, as shown by the fluorescence decay kinetics (Figure 2). These data confirm that EH possesses substantial intrinsic antioxidant activity.

### 2.2. EH Protects HepG2 Cells Against Fatty Acid-Induced Membrane Damage

To determine the safety range of EH in an in vitro model of hepatic steatosis, adherent cell biomass was initially assessed using the sulforhodamine B (SRB) assay. HepG2 cells were exposed to a mixture of oleic and palmitic acids for 24 h to induce lipid overload, followed by treatment with increasing concentrations of EH (50–250 µg/mL) for an additional 24 h.

**Figure 3:**
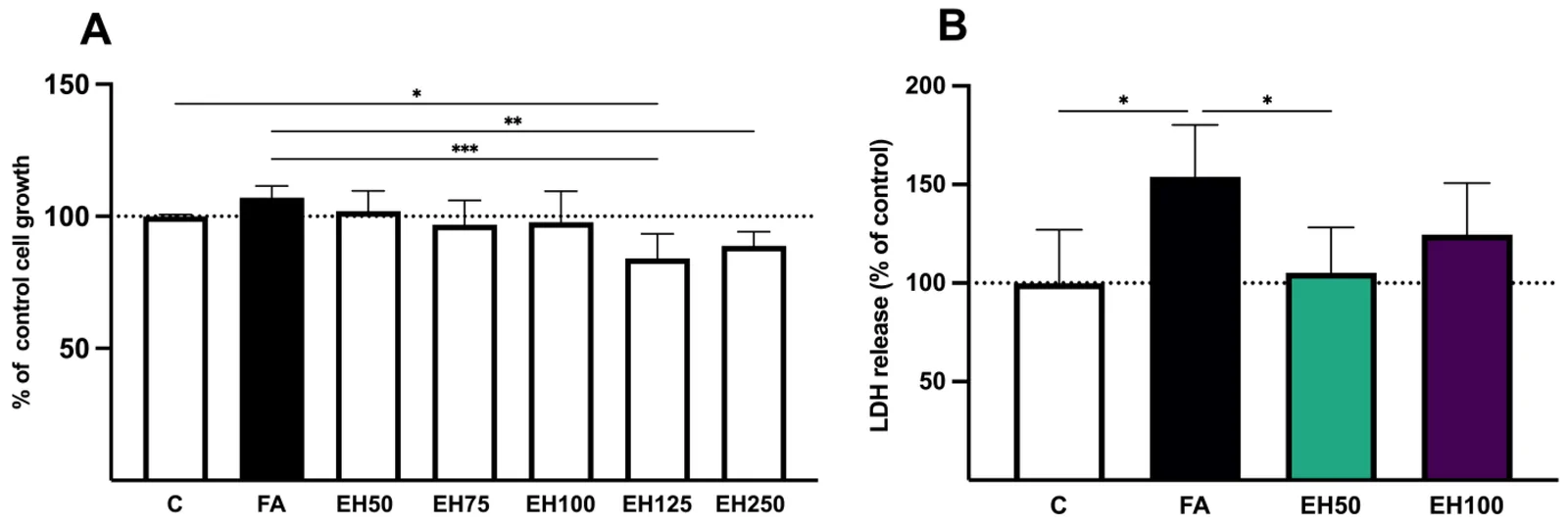
Effect of *H. speciosa* leaf aqueous extract (EH) on adherent cell biomass and plasma membrane integrity in HepG2 cells with fatty acid-induced lipid accumulation. HepG2 cells were exposed to fatty acids for 24 h, followed by treatment with EH for an additional 24 h. (**A**) Cell biomass assessed by the sulforhodamine B (SRB) assay after treatment with increasing concentrations of EH (50–250 µg/mL). (**B**) Plasma membrane integrity assessed by lactate dehydrogenase (LDH) release into the culture supernatant after treatment with EH at 50 and 100 µg/mL. Data are presented as mean ± standard deviation from at least three independent experiments performed in triplicate. * *p* < 0.05; ** *p* < 0.01; *** *p* < 0.001.

SRB staining showed no significant differences between the control (C) and fatty acid-treated (FA) groups. Similarly, treatment with EH at 50, 75, and 100 µg/mL resulted in SRB values comparable to those of both the C and FA groups. Although EH at 125 and 250 µg/mL maintained SRB values above 70% of the control, both concentrations resulted in significantly lower values than the FA group (Figure 3A). Because the SRB assay quantifies cellular protein, these results reflect changes in adherent cell biomass and should not be interpreted as a direct measure of cell viability.

Based on the initial screening, concentrations of 50 and 100 µg/mL (EH50 and EH100) were selected for subsequent experiments because they preserved cell biomass at levels comparable to both the C and FA groups and also showed pronounced radical-scavenging activity in the ORAC assay.

To further assess cellular injury, plasma membrane integrity was evaluated by measuring LDH release into the culture supernatant. FA exposure significantly increased LDH release compared with the C group, indicating fatty acid-induced membrane damage (Figure 3B). Treatment with EH50 effectively attenuated this damage, significantly reducing LDH release back to control levels. In contrast, EH100 treatment showed no significant changes compared to either the control or FA groups.

### 2.3. EH Attenuates Fatty Acid-Induced Lipid Accumulation in HepG2 Cells

**Figure 4:**
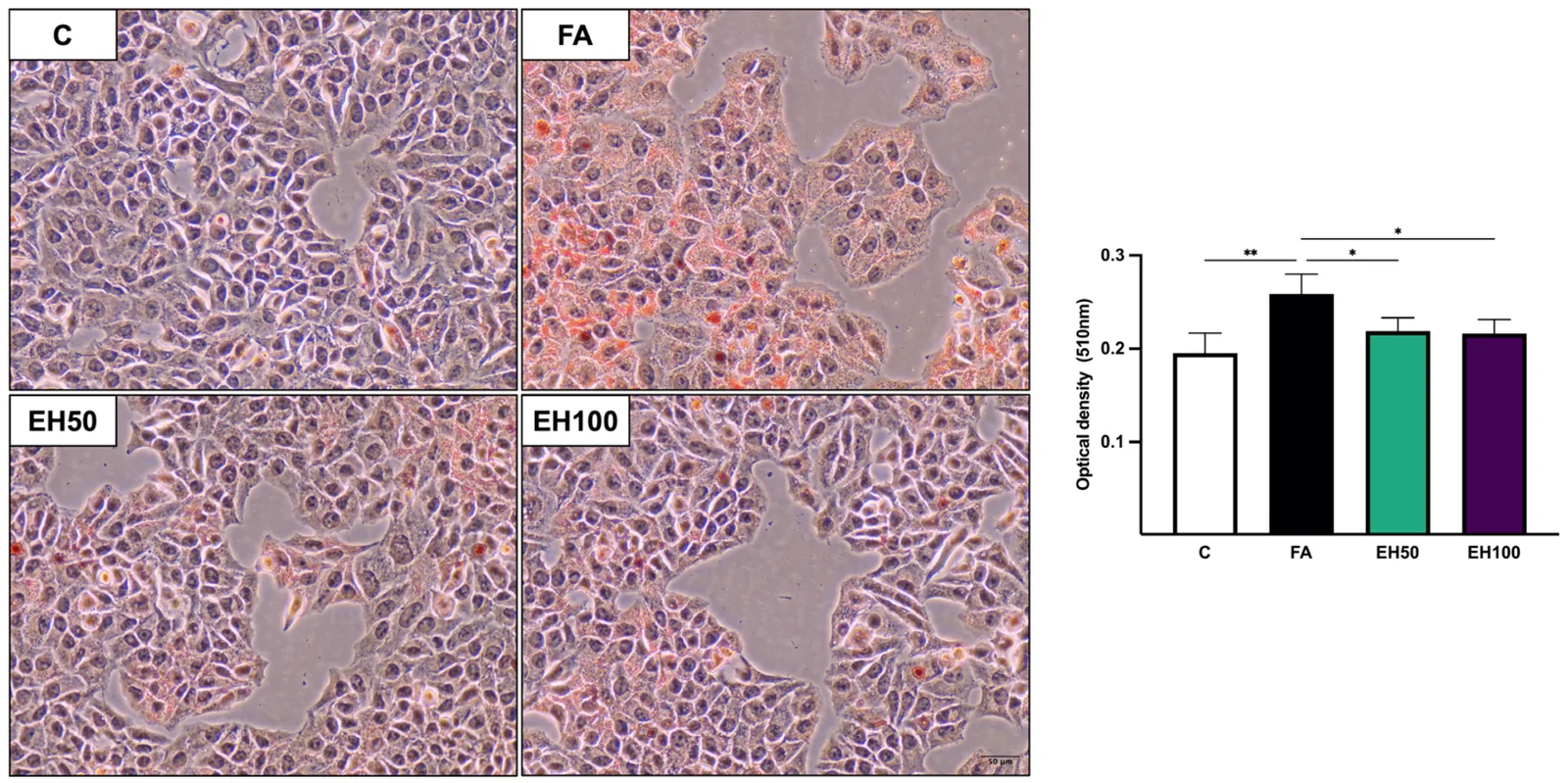
Effect of *H. speciosa* leaf aqueous extract (EH) on intracellular lipid content in HepG2 cells with fatty acid-induced lipid accumulation. Representative photomicrographs of Oil Red O (ORO) and hematoxylin staining (200× magnification, scale bar: 50 μm) and quantification of intracellular lipid content measured by absorbance at 510 nm. HepG2 cells were exposed to fatty acids for 24 h, followed by treatment with EH (50 and 100 µg/mL) for an additional 24 h. Data are presented as mean ± standard deviation from at least three independent experiments performed in triplicate. * *p* < 0.05; ** *p* < 0.01.

The extent of the effect of EH on intracellular lipid accumulation was quantified by Oil Red O staining. As expected, FA exposure induced a significant increase in lipid droplet content, demonstrating effective in vitro steatosis induction (Figure 4). Notably, EH50 and EH100 exhibited a significant reduction in lipid overload when compared to the FA group, suggesting a hypolipidemic effect.

### 2.4. EH Improves Redox Homeostasis in HepG2 Cells with Lipid Overload

To determine whether the EH-mediated attenuation of lipid accumulation was accompanied by restoration of cellular redox balance, key parameters of cellular redox homeostasis were analyzed.

**Figure 5:**
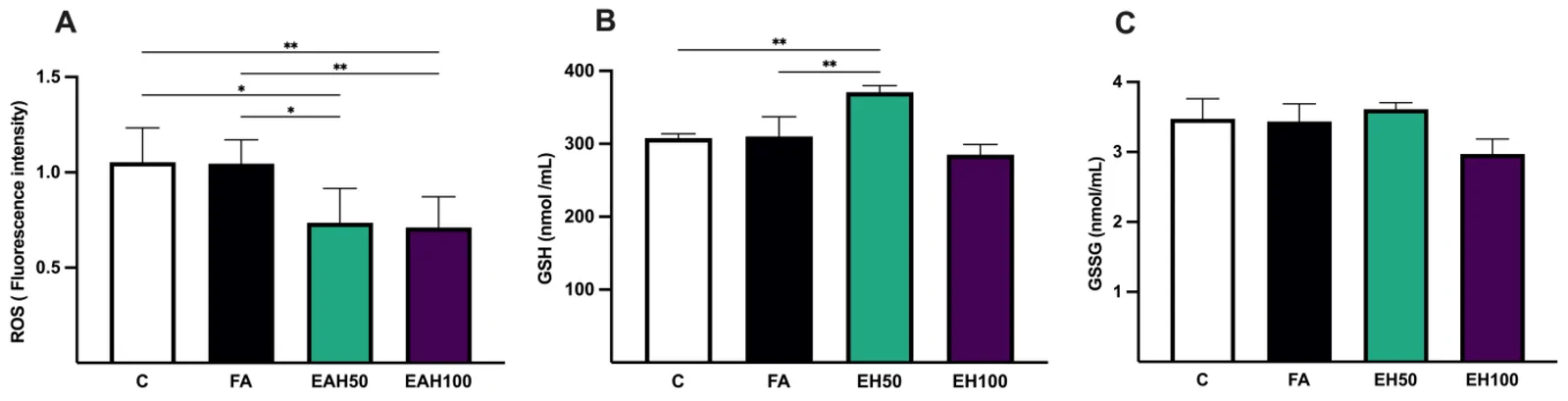
Effect of *H. speciosa* leaf aqueous extract (EH) on redox homeostasis in HepG2 cells with fatty acid-induced lipid accumulation. (**A**) Intracellular reactive oxygen species (ROS) levels, (**B**) total glutathione (GSH), and (**C**) oxidized glutathione (GSSG) levels. HepG2 cells were exposed to fatty acids for 24 h, followed by treatment with EH (50 and 100 µg/mL) for an additional 24 h. Data are presented as mean ± standard deviation from at least three independent experiments performed in triplicate. * *p* < 0.05; ** *p* < 0.01.

Intracellular ROS quantification revealed no significant differences between the control and FA groups. However, treatment with EH50 and EH100 significantly decreased ROS generation compared with both the C and FA groups (Figure 5). Total glutathione levels were not significantly altered by fatty acid exposure alone. Interestingly, EH50 treatment significantly increased total GSH content compared with both the C and FA groups, whereas EH100 maintained levels comparable to the control. Oxidized glutathione (GSSG) content showed no significant difference among the experimental groups.

**Figure 6:**
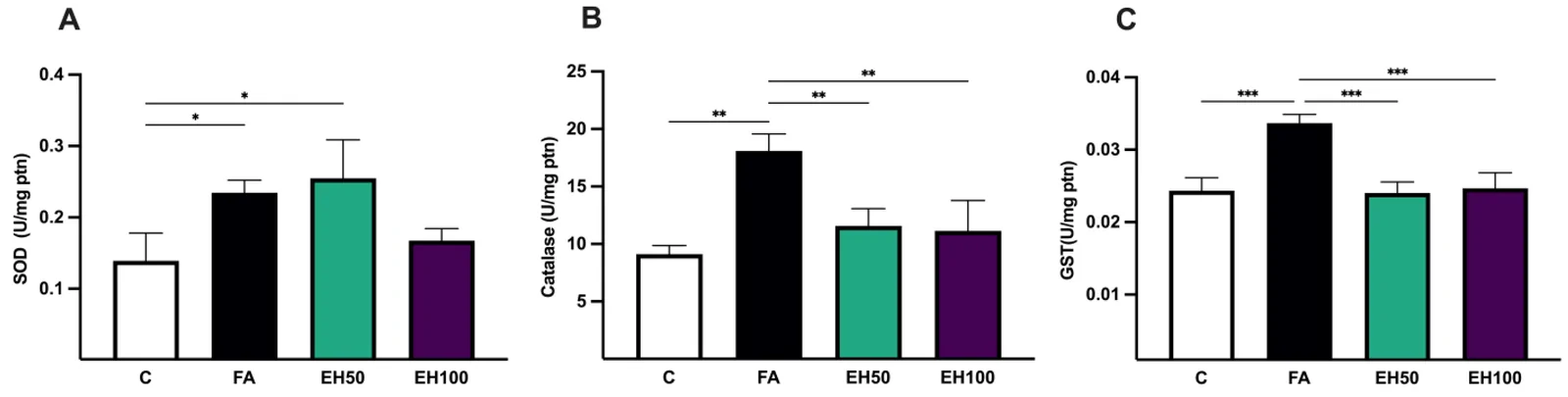
Effect of *H. speciosa* leaf aqueous extract (EH) on antioxidant enzyme activities in HepG2 cells with fatty acid-induced lipid accumulation. Activities of (**A**) superoxide dismutase (SOD), (**B**) catalase (CAT), and (**C**) glutathione S-transferase (GST) were measured in HepG2 cells exposed to fatty acids for 24 h, followed by treatment with EH (50 and 100 µg/mL) for an additional 24 h. Data are presented as mean ± standard deviation from at least three independent experiments performed in triplicate. * *p* < 0.05; ** *p* < 0.01; *** *p* < 0.001.

Assessment of endogenous enzymatic defenses showed distinct modulatory patterns under lipid overload and EH treatments (Figure 6). Superoxide dismutase (SOD) activity was significantly elevated in the FA and EH50 groups relative to control, whereas the EH100 group showed no significant difference. Conversely, catalase (CAT) and glutathione S-transferase (GST) demonstrated similar outcomes. Activities of both enzymes increased significantly in response to FA. Treatments with EH50 and EH100 suppressed this FA-induced elevation, restoring enzymatic activity back to levels not statistically different from the control group.

### 2.5. EH Affects Sphingolipid-Related Metabolic Features in HepG2 Cells with Lipid Overload

**Figure 7:**
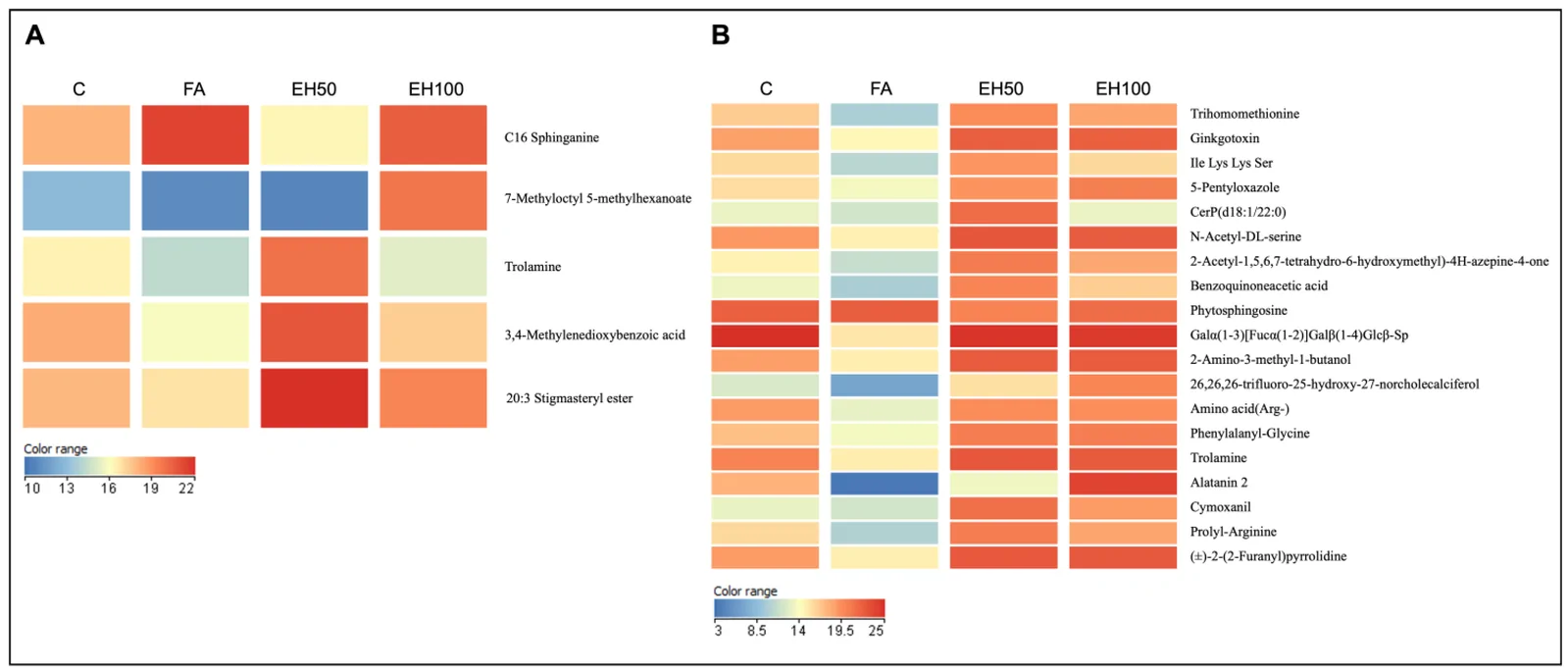
Effect of *H. speciosa* leaf aqueous extract (EH) treatment on intracellular and extracellular metabolomic profiles in HepG2 cells with fatty acid-induced lipid accumulation. HepG2 cells were exposed to fatty acids for 24 h, followed by treatment with EH (50 and 100 µg/mL) for an additional 24 h. Untargeted metabolomic profiling was performed using high-performance liquid chromatography coupled to electrospray ionization quadrupole time-of-flight mass spectrometry (HPLC-ESI-QTOF-MS). Heatmaps show differentially abundant metabolic features annotated as (**A**) intracellular and (**B**) extracellular metabolites based on accurate mass matching against the METLIN database. Data were obtained from six independent experiments performed in triplicate. Red indicates higher relative abundance, whereas blue indicates lower relative abundance.

To further characterize the metabolic alterations associated with lipid overload and the protective effects of EH, untargeted metabolomic profiling was conducted on both the intracellular metabolome and exometabolome (culture medium) of HepG2 cells (Figure 7).

Untargeted HPLC-MS profiling initially detected 679 metabolic features in cell lysates. Following a 100% frequency filter, 23 features were retained, five of which exhibited significant differential abundance among the experimental groups. These features were subsequently annotated based on accurate mass matching against the METLIN database as C16 sphinganine (dihydrosphingosine), 7-methyloctyl 5-methylhexanoate, trolamine, 3,4-methylenedioxybenzoic acid (piperonylic acid), and stigmasteryl ester 20:3 (Figure 7). The FA group showed a markedly higher relative abundance of intracellular C16 sphinganine compared to the control group, whereas its abundance was lower in the EH50 group compared to the FA group. Piperonylic acid and stigmasteryl ester 20:3 also displayed higher relative abundance in the EH50 group compared to the FA group. In contrast, 7-methyloctyl 5-methylhexanoate was the only annotated molecular feature that differed significantly between the EH100 and FA groups.

In parallel, the analysis of the culture medium provided complementary insights into extracellular metabolite alterations. A total of 607 metabolic features were initially detected, of which 31 were retained after applying the 100% frequency filter. Among these, 19 showed significant differences in abundance among the experimental groups and were annotated as trihomomethionine, ginkgotoxin, Ile Lys Ser, 5-pentyloxazole, CerP(d18:1/22:0), N-acetyl-DL-serine, 2-acetyl-1,5,6,7-tetrahydro-6-hydroxy-7-(hydroxymethyl)-4H-azepine-4-one, benzoquinoneacetic acid, phytosphingosine; Galα(1-3)[Fucα(1-2)]Galβ(1-4)Glcβ-Sp, 2-amino-3-methyl-1-butanol; 6,26,26-trifluoro-25-hydroxy-27-norcholecalciferol, amino acid (Arg-), phenylalanyl-glycine; trolamine, alatanin 2, cymoxanil, prolyl-Arginine, and (±)-2-(2-Furanyl)pyrrolidine. Among the most biologically relevant features, ceramide phosphate CerP(d18:1/22:0) showed markedly higher relative abundance in the culture medium of the EH50 group compared with the FA group, whereas phytosphingosine showed significantly lower relative abundance. These changes suggest alterations in sphingolipid metabolic homeostasis.

## 3. Discussion

The present study characterized the phytochemical composition of EH and provided evidence of its lipid-lowering and antioxidant effects in HepG2 cells with fatty acid-induced lipid accumulation. Notably, untargeted metabolomic profiling suggests coordinated changes in sphingolipid-related metabolite features, associated with the attenuation of lipotoxicity, providing novel insights into the metabolic pathways potentially involved.

The selection of an aqueous extract was based on the traditional medicinal use of *H. speciosa*, which commonly involves water-based infusions, providing an ethnopharmacological rationale for the extraction method employed in this study. Phytochemical characterization revealed the presence of phenolic acids, flavonoids, terpenoids, organic acids, and oxygenated fatty acids in EH, including catechin, epicatechin, rutin, and a glycosylated kaempferol derivative. This chemical profile, together with the high phenolic and flavonoid contents, highlights the chemical complexity of the extract and is consistent with previous studies describing the phytochemical composition of *H. speciosa* [26,27,38,40]. Phenolic compounds are widely recognized for their antioxidant properties and ability to modulate metabolic processes [14,41,42] and may therefore contribute to the protective effects of EH on cellular damage, lipid accumulation, and oxidative stress observed in the present study.

The combined assessment of cell biomass and membrane integrity provides complementary information on cellular responses to experimental treatment [43]. The apparent difference between the SRB and LDH release findings may reflect the distinct nature and sensitivity of each assay. The SRB assay is based on the binding of the dye to cellular proteins and therefore primarily reflects the biomass of cells remaining adherent after treatment, rather than directly assessing cell death [44]. In contrast, LDH is a cytosolic enzyme released into the extracellular medium after loss of plasma membrane integrity and is therefore a sensitive indicator of cell membrane damage [45]. Thus, preserved SRB staining following fatty acid exposure does not exclude the occurrence of cellular injury. Accordingly, the increased LDH release observed in the FA group indicated impaired cell membrane integrity. Previous studies reported that lipotoxicity induced by fatty acids leads to cell damage [46]. Treatment with EH50 significantly restored LDH release to levels comparable to those in the control group, suggesting a potential protective effect.

HepG2 cells exposed to palmitic and oleic acids showed increased intracellular lipid content, consistent with previous studies that reproduced hepatic steatosis in vitro using similar experimental conditions [11]. Treatment with EH50 and EH100 significantly reduced lipid accumulation. Polyphenols and other plant-derived bioactive compounds have been reported to modulate pathways involved in lipid metabolism [14,47]. Activation of AMP-activated protein kinase (AMPK) has been associated with reduced hepatic lipogenesis and inhibition of lipogenic regulators, including sterol regulatory element-binding protein 1c (SREBP-1c) [48,49,50]. Similarly, an epigallocatechin-3-gallate-rich green tea extract reduced hepatic lipid accumulation in a high-fat diet model, an effect associated with SIRT1 and AMPK activation and decreased expression of lipogenesis-related enzymes [51]. Therefore, phenolic compounds annotated in EH, such as catechin, epicatechin, rutin, and kaempferol, may have contributed to the observed effect.

Regarding oxidative stress, intracellular ROS levels did not differ between the C and FA groups, whereas treatment with EH at both concentrations significantly reduced the intracellular ROS levels. It should be considered that the method used provides an endpoint measurement; therefore, the absence of a difference between the C and FA groups does not exclude the possibility of transient changes at earlier stages of the experiment. Oxidative stress is a multifactorial process arising from an imbalance between reactive species production and the capacity of antioxidant systems; thus, it cannot be characterized solely by ROS quantification at a single time point [52,53,54]. Accordingly, these findings should be interpreted in conjunction with other redox status biomarkers.

Glutathione is a major intracellular antioxidant that plays a central role in maintaining cellular redox homeostasis [55,56]. In the present study, EH50 treatment significantly increased total glutathione levels without significantly affecting oxidized glutathione, consistent with an increase in the reduced glutathione pool. Since intracellular GSH levels are regulated by de novo synthesis, enzymatic recycling of GSSG to GSH, and its utilization in detoxification reactions, the increase in GSH following EH50 treatment may reflect changes in glutathione metabolism. The involvement of the nuclear factor erythroid 2-related factor 2 (Nrf2) pathway represents a plausible hypothesis, as this transcription factor regulates antioxidant gene expression. Nrf2 transcriptional targets include glutamate–cysteine ligase (GCL), a key enzyme involved in glutathione biosynthesis [57,58]. Phenolic compounds are known to activate the Nrf2 pathway, thereby contributing to the maintenance of cellular redox homeostasis [14].

Our results showed increased SOD, CAT, and GST activities in the FA group, suggesting an adaptive antioxidant response to lipid overload and redox imbalance [4], which may have contributed to limiting the increase in ROS concentrations. Treatment with EH50 and EH100 reduced CAT and GST activity to levels comparable to those observed in the control group. Given that CAT catalyzes the decomposition of hydrogen peroxide and GST participates in the detoxification of electrophilic compounds and lipid peroxidation products, the normalization of their activities, together with reduced lipid accumulation and lower intracellular ROS levels, is consistent with a reduced oxidative burden. The phenolic constituents of EH may have contributed to these effects, given the direct antioxidant capacity demonstrated by the ORAC assay. These findings are consistent with the reported effects of phenolic compounds on metabolic and oxidative processes [14,41,42] and support previous studies describing the biotechnological potential and antioxidant, hepatoprotective, and hypolipidemic activities of *H. speciosa* extracts [26,27,29,38]. However, the observed effects cannot be attributed to any specific EH constituent. Given the complex and unfractionated nature of the extract, these biological effects may result from additive or synergistic interactions among its constituents.

Global metabolomic profiling revealed differential abundance of metabolic features associated with sphingolipid metabolism, providing additional insight into the cellular response to fatty acid overload and EH treatment. Among the annotated metabolites, C16 sphinganine showed increased intracellular abundance following fatty acid exposure. Sphinganine (dihydrosphingosine) is a central intermediate in the de novo sphingolipid biosynthetic pathway, generated downstream of serine and palmitoyl-CoA and subsequently N-acylated by ceramide synthases to form dihydroceramides, which are further converted into ceramides. Thus, the increased abundance of C16 sphinganine following fatty acid exposure may indicate enhanced flux or perturbation of de novo sphingolipid metabolism under conditions of lipid overload. This interpretation is consistent with the established association between dysregulated sphingolipid metabolism, particularly ceramide accumulation, and several mechanisms of lipotoxicity, including endoplasmic-reticulum stress, mitochondrial dysfunction, oxidative stress, activation of stress-responsive signaling pathways such as JNK, and apoptotic signaling [3,59,60]. Increased sphinganine levels have been reported in an in vivo model of MASLD and were attenuated by lipoic acid treatment [61], further supporting a link between alterations in sphingolipid metabolism and metabolic liver injury.

Remarkably, EH50 treatment reduced the intracellular abundance of C16 sphinganine. When considered together with the decreased lipid accumulation and reduced LDH release, this finding suggests that attenuation of fatty acid-induced lipotoxicity by EH may be associated with modulation of sphingolipid metabolism.

Additional changes in the extracellular metabolomic profile provide further evidence of alterations in sphingolipid-related metabolites following EH treatment. EH50 was associated with lower extracellular abundance of the molecular feature annotated as phytosphingosine, together with increased extracellular abundance of CerP(d18:1/22:0). Ceramide-1-phosphate (CerP) is a phosphorylated sphingolipid metabolite generated from ceramide and is involved in lipid signaling, membrane dynamics, inflammatory responses, and cell survival [62]. Although the biological significance of increased extracellular abundance of CerP(d18:1/22:0) remains to be established, this finding may suggest altered ceramide processing and/or redistribution toward extracellular compartments associated with EH treatment.

This pattern may also be relevant to the cellular redox responses. Ceramide kinase (CERK) catalyzes the phosphorylation of ceramide to form CerP, which can be released into the extracellular compartment [62]. CERK/C1P signaling has been associated with antioxidant protection through NRF2 activation and nuclear translocation [63]. Therefore, the observed sphingolipid-associated changes raise the possibility that EH promotes a shift in homeostasis away from the accumulation of potentially lipotoxic intracellular intermediates. Such remodeling may contribute to increased antioxidant capacity, lower ROS levels, and reduced cytotoxicity following EH treatment. Further studies combining validated metabolite annotations using authentic reference standards, targeted lipidomic analysis, and functional assessment of the CERK/C1P–NRF2 axis are needed to establish a mechanistic link between these findings.

Although both EH50 and EH100 exerted protective effects against fatty acid-induced lipotoxicity, a consistent concentration-dependent response was not observed across the evaluated parameters. Previous studies have demonstrated biphasic concentration–response relationships for plant-derived bioactive compounds, in which higher concentrations can inhibit or even reverse the effects observed at lower concentrations [64,65].

Taken together, the findings of the present study demonstrate that EH reduces intracellular lipid accumulation while modulating redox homeostasis in HepG2 cells with lipid overload. Untargeted metabolomic profiling further suggests that a remodeling of sphingolipid homeostasis may be associated with the protective effects of EH against fatty acid-induced lipotoxicity. These findings expand current knowledge of the biological effects of *H. speciosa* leaf aqueous extract and strengthen evidence for the therapeutic potential of the species as a source of bioactive compounds relevant to metabolic disorders, paving the way for further studies aimed at elucidating the underlying molecular mechanisms.

## 4. Materials and Methods

### 4.1. Sample Collection and Extract Preparation

*Hancornia speciosa* leaves were collected in Patrocínio, Minas Gerais, Brazil (18°55′16.9″ S; 46°59′51.2″ W). A voucher specimen was deposited at the Uberlandense Herbarium, Federal University of Uberlândia, Minas Gerais, Brazil (accession number 90909).

The leaves were then frozen, freeze-dried, and pulverized. The aqueous extract was prepared by infusing the plant material in water at a 1:10 (*w*/*v*) ratio in a water bath at 72 °C for 10 min. The resulting extract was vacuum filtered, freeze-dried, and stored at 4 °C until use.

### 4.2. Phytochemical Characterization and Antioxidant Capacity

To comprehensively characterize the chemical profile and evaluate the in vitro antioxidant properties of the aqueous extract of *H. speciosa*, a complementary approach combining high-resolution mass spectrometry and spectrophotometric/fluorometric assays was employed. Untargeted phytochemical profiling was performed by high-performance liquid chromatography coupled with electrospray ionization tandem mass spectrometry (HPLC-ESI-MS/MS).

For HPLC-ESI-MS/MS, samples were solubilized in a water–methanol (7:3, *v*/*v*) solution at a concentration of 5 mg mL^−1^, filtered through 0.22 μm nylon membrane filters, and injected into an Agilent Infinity 1260 liquid chromatograph coupled to an Agilent 6520B high-resolution quadrupole time-of-flight (Q-TOF) mass spectrometer with an electrospray ionization (ESI) source. Chromatographic separation was performed on an Agilent InfinityLab Poroshell HPH-C18 column (2.1 × 50 mm, 2.7 μm) at a flow rate of 400 μL min^−1^. The mobile phase consisted of (A) water acidified with 0.1% (*v*/*v*) formic acid and (B) methanol. The elution gradient was programmed as follows: 10% B (0 min), 10–98% B (0–15 min), and 100% B (15–17 min). The ionization parameters were as follows: nebulizer pressure, 58 psi; drying gas flow, 8 L min^−1^ at 220 °C; and capillary voltage, 4.5 kV. Tandem mass spectrometry (MS/MS) analyses were performed using collision energies of 5, 10, 15, 20, 25 eV, and 30 eV. Data were processed using MassHunter^®^ software (version 10.0), which proposed molecular formulas based on the *m*/*z* values. Compound annotation was performed considering high-resolution *m*/*z* values (mass error < 10 ppm), MS/MS fragmentation patterns, and comparisons with literature data and specialized databases, including PubChem, HMDB, and Metabolomics Workbench.

The ORAC assay was performed according to an established method [66], with minor modifications. In a 96-well microplate, EH (5, 10, 50, and 100 µg/mL), vehicle control, or the reference antioxidant Trolox was incubated with fluorescein in phosphate-buffered saline (PBS) for 15 min. The reaction was initiated by the addition of AAPH, and fluorescence was monitored at excitation and emission wavelengths of 485 and 535 nm, respectively, at 1 min intervals for 60 min using a VICTOR™ X3 fluorometer (PerkinElmer, Waltham, MA, USA). The highest fluorescence reading from the AAPH-free wells was used as the reference point for fluorescence measurements. The area under the fluorescence decay curve (AUC) was calculated for each sample. A standard curve was constructed by plotting AUC values against Trolox concentrations. The antioxidant capacity of EH was expressed as µmol TE/g freeze-dried extract.

Total phenolic content was determined using the Folin–Ciocalteu method according to Georgé et al. [67], with gallic acid used as the standard for the calibration curve. Results are expressed as milligrams of gallic acid equivalents per gram of freeze-dried extract (mg GAE/g freeze-dried extract). Aluminum chloride-reactive flavonoid content was determined colorimetrically according to Hossain and Rahman [68], using quercetin as the standard. Results are expressed as milligrams of quercetin equivalents per gram of freeze-dried extract (mg QE/g). All analyses were performed in triplicate.

### 4.3. Cell Culture

HepG2 cells were obtained from the Rio de Janeiro Cell Bank (BCRJ) and maintained in low-glucose Dulbecco’s modified Eagle’s medium (DMEM) supplemented with 10% fetal bovine serum (FBS), 100 U/mL penicillin, and 100 μg/mL streptomycin at 37 °C in a humidified atmosphere containing 5% CO_2_. Cells were subcultured every 2–3 days using 0.25% trypsin–EDTA. Cells between passages 5 and 10 were used throughout the experiments. All experiments were performed using cultures at 70–80% confluence.

### 4.4. Induction of Lipid Accumulation in HepG2 Cells and EH Treatment

For cell-based assays, HepG2 cells were seeded at a density of 2 × 10^5^ cells/well in 6-well plates or 3 × 10^3^ cells/well in 96-well plates and allowed to adhere for 24 h. Subsequently, the cells were incubated in serum-free medium for 24 h and then exposed for an additional 24 h to a mixture of oleic acid (OA) and palmitic acid (PA) at a 2:1 molar ratio, corresponding to final concentrations of 1.33 mM OA and 0.67 mM PA (2.0 mM total fatty acids). The fatty acid mixture was conjugated to 4.5% fatty acid-free bovine serum albumin (BSA), as described by Arruda et al. [11]. Control cells were incubated under the same conditions with medium containing 4.5% BSA without fatty acids. Following the 24 h fatty acid exposure, cells were washed and subsequently incubated for an additional 24 h with fresh medium containing different concentrations of EH. All experiments were performed in triplicate in at least three independent experiments.

### 4.5. Adherent Cell Biomass and LDH Release

Cell biomass was assessed using the sulforhodamine B (SRB) assay (Invitrogen^®^, Waltham, MA, USA), as previously described [44]. Briefly, cells were fixed with 50% trichloroacetic acid (TCA), stained with 0.04% SRB, and washed with 1% acetic acid. The protein-bound dye was solubilized in 10 mM Tris base, and the resulting absorbance was measured at 510 nm. Results are expressed as a percentage of control growth.

Lactate dehydrogenase (LDH) release was assessed using the CyQUANT^™^ LDH cytotoxicity assay kit (Thermo Fisher Scientific, Waltham, MA, USA). Following experimental treatments, culture supernatants were collected, and the assay was performed according to the manufacturer’s instructions. A maximum LDH release control was included by lysing cells with Triton X-100, as specified in the CyQUANT™ LDH Cytotoxicity Assay Kit protocol. Absorbance was measured at 490 and 680 nm, and % of LDH release was calculated relative to the control group.

### 4.6. Intracellular Lipid Content

Intracellular lipid accumulation was assessed using Oil Red O (ORO) staining, as described by Arruda et al. [11]. Cells were fixed with 4% paraformaldehyde for 15 min and subsequently incubated with a 60% ORO solution for 50 min. After washing, the retained dye was eluted with 60% isopropanol, and absorbance was measured at 510 nm. For morphological analysis, cell nuclei were counterstained with hematoxylin and visualized using an optical microscope (EVOS XL Core, Thermo Fisher Scientific, Waltham, MA, USA) at 200× magnification.

### 4.7. Cellular Redox Homeostasis

To investigate the effects of fatty acid overload and EH treatments on redox homeostasis in HepG2 cells, intracellular ROS levels and endogenous antioxidant defenses were evaluated.

Intracellular ROS levels were quantified using the Image-iT^™^ LIVE Green Reactive Oxygen Species (ROS) Detection Kit (Thermo Fisher Scientific, Waltham, MA, USA), based on the fluorogenic probe 5-(and-6)-carboxy-2’,7’-dichlorodihydrofluorescein diacetate (carboxy-H_2_DCFDA). Following treatment, cells were incubated with the fluorophore working solution (25 μM) for 30 min at 37 °C in the dark. As a positive control for oxidative stress induction, cells were treated with tert-butyl hydroperoxide (TBHP, 100 μM) for 90 min at 37 °C under 5% CO_2_. After washing, fluorescence intensity was measured using a microplate reader at excitation and emission wavelengths of 495 nm and 529 nm, respectively.

Total glutathione (GSH) and oxidized glutathione (GSSG) levels were determined using an enzymatic recycling assay [69] with modifications. Treated cells were homogenized in an extraction buffer containing Triton X-100, sulfosalicylic acid, 0.1 M potassium phosphate buffer (KPE), and 5 mM disodium EDTA. After centrifugation and freeze–thaw cycles, the cell lysates were incubated with a working mixture containing KPE, 5,5′-dithiobis(2-nitrobenzoic acid) (DTNB), and glutathione reductase, followed by the addition of NADPH. Absorbance was monitored at 405 nm at 30 s intervals for 5 min. For GSSG determination, 2-vinylpyridine was added for derivatization for 1 h, and the samples were analyzed as described above. Reduced and oxidized glutathione standards were used to construct calibration curves. The results are expressed as nmol/mL.

Glutathione S-transferase (GST) activity was determined based on the conjugation of reduced glutathione to 1-chloro-2,4-dinitrobenzene (CDNB), according to Habig et al. [70]. Following cell lysis and centrifugation, the supernatant was incubated with a phosphate buffer, reduced GSH, and CDNB. Conjugate formation was monitored at 340 nm for 5 min. GST activity is expressed as µmol/mg protein.

Superoxide dismutase (SOD) activity was determined as described by Madesh and Balasubramanian [71], based on pyrogallol autoxidation and superoxide anion-mediated reduction of 3-(4,5-dimethylthiazol-2-yl)-2,5-diphenyltetrazolium bromide (MTT). Following incubation with MTT and pyrogallol, the reactions were terminated by the addition of dimethyl sulfoxide (DMSO), and absorbance was measured at 570 nm using a microplate reader. SOD activity was calculated based on the ability of the enzyme to inhibit formazan formation. One unit of SOD activity was defined as the amount of enzyme required to inhibit formazan formation by 50%.

Catalase (CAT) activity was determined using the kinetic method described by Grilo et al. [72], with modifications. Cell lysates were prepared in 50 mM phosphate buffer (pH 7.8) containing 1% Triton X-100 and centrifuged at 12,000× *g* for 10 min at 4 °C. The assay was performed in a 96-well UV microplate containing 20 µL of sample, 180 µL of phosphate buffer, and 100 µL of 50 mM H_2_O_2_. The decrease in absorbance was monitored at 240 nm at 15 s intervals for 2.5 min. Enzyme activity was normalized to the protein concentration and expressed as U/mg protein.

Total protein concentration was determined using the Bradford assay with bovine serum albumin (BSA) as the standard [73]. Samples and standards were incubated with Bradford reagent in 96-well plates for 5 min, and absorbance was measured at 595 nm using a microplate reader. Protein concentrations were calculated by interpolation from the standard curve.

### 4.8. Untargeted Metabolomic Analysis

Untargeted metabolomic profiling was performed to detect molecular features differentially abundant among the experimental groups.

For intracellular metabolite extraction, cells were collected and centrifuged at 3000× *g* for 5 min. The resulting cell pellet was mixed with 1000 µL of spectroscopic-grade methanol and vortexed for 5 min at 1300× *g*. The samples were subsequently centrifuged at 13,000× *g* for 15 min, and the resulting supernatants were concentrated using a vacuum concentrator for 120 min. For extracellular metabolite analysis, 100 µL of cell culture medium was collected and concentrated using a vacuum concentrator for 120 min. Samples were then resuspended in spectroscopic-grade methanol and filtered through a 0.22 µm syringe filter. Samples from at least six independent biological experiments were analyzed, with each biological replicate measured in technical triplicate.

High-performance liquid chromatography-mass spectrometry (HPLC-MS) analysis was performed using an Agilent Infinity 1260 HPLC system coupled to an Agilent 6520B high-resolution quadrupole time-of-flight (Q-TOF) mass spectrometer equipped with an electrospray ionization (ESI) source. Chromatographic separation was carried out on an Agilent Poroshell C18 column (2.1 mm internal diameter × 50 mm length; 2.7 μm particle size) maintained at 35 °C. The mobile phase consisted of water containing 0.1% (*v*/*v*) formic acid (mobile phase A) and methanol (mobile phase B), with the following gradient: 2% B at 0 min, 2–98% B from 0 to 6 min, and 98% B from 6 to 12 min. The ionization parameters were as follows: nebulizer pressure, 25 psi; drying gas flow rate, 8 L/min at 220 °C; and capillary voltage, 4.5 kV. Collision energy for fragmentation was calculated according to the following equation: Collision Energy (eV) = [3 × (*m*/*z*)/100] + 5. Mass spectra were acquired in positive ionization mode over an *m*/*z* range of 55–1500, with data recorded in both centroid and profile formats.

Mass spectral data were extracted using MassHunter Qualitative Analysis software (version 10.1) and converted to Compound Exchange Format (.CEF) files. The resulting files were subsequently processed using the MassHunter Mass Profiler Professional (MPP) software (version B.15.1). Filtering parameters included a minimum absolute abundance threshold of 5000 counts, considering all allowable charge states. Retention time and mass tolerance were set to 0.15 min and 15 ppm ± 2 mDa, respectively. Only molecular features detected in 100% of the samples within at least one experimental group were retained for subsequent analysis.

Metabolite annotation was performed using the METLIN 2019 database integrated into MPP. Molecular features were putatively annotated based on accurate mass, within the mass tolerance specified above. No chemical standards were analyzed; these assignments correspond to level 2, putatively annotated compounds, according to the Metabolomics Standards Initiative (MSI) criteria [74].

For statistical analysis, technical replicates of each independent biological experiment were averaged, and quantitative data were log2-transformed. Overall differences among experimental groups were assessed using the Kruskal–Wallis test, followed by pairwise comparisons with the Mann–Whitney U test. Molecular features were considered differentially abundant when *p* < 0.05 and fold change ≥ 2.0.

### 4.9. Statistical Analysis

Data distribution was assessed using the Shapiro–Wilk test, and homogeneity of variances was evaluated using the Brown–Forsythe test. Differences among experimental groups were analyzed by one-way ANOVA, followed by Tukey’s post hoc multiple-comparison test. Differences were considered statistically significant at *p* < 0.05. All statistical analyses were performed using GraphPad Prism version 10.3.1 (GraphPad Software, San Diego, CA, USA).

## Figures and Tables

**Table 1:** Compounds annotated in aqueous extract of *H. speciosa* leaves by HPLC-ESI-MS/MS in negative mode.

*t*_R_ (Min)	Experimental Mass [M − H]^−^ (*m*/*z*)	Exact Mass [M − H]^−^ (*m*/*z*)	Error (ppm)	Fragment Ions MS/MS (*m*/*z*)	Molecular Formula	Annotated Compounds	Reference
0.45	191.0562	191.0561	0.52	20 eV: 173.0477, 127.0408, 111.0091, 93.0364, 85.0305	C_7_H_12_O_6_	Quinic acid	[30]
0.50	133.0141	133.0142	−0.75	15 eV: 115.0081, 89.0276, 71.0188	C_4_H_6_O_5_	Malic acid	[30]
1.63	353.0882	353.0878	1.13	20 eV: 191.0562, 179.0356, 135.0487	C_16_H_18_O_9_	Caffeoylquinic acid isomer I	[31,32]
2.06	577.1350	577.1351	−0.17	20 eV: 451.1056, 425.0877, 407.0728, 289.0717, 245.0795, 125.0250	C_30_H_26_O_12_	(epi)catechin-(epi)catechin	[33]
2.27	337.0928	337.0929	−0.29	15 eV: 191.0629, 173.0505, 163.0458, 119.0558	C_16_H_18_O_8_	Coumaroylquinic acid isomer I	[34]
2.53	289.0721	289.0718	1.04	15 eV: 245.0898, 205.0600, 179.0393, 151.0462, 137.0298, 125.0325, 109.0368	C_15_H_14_O_6_	Catechin	[35,36]
2.70	353.0878	353.0878	0.00	20 eV: 191.0629, 161.0303, 135.0528	C_16_H_18_O_9_	Caffeoylquinic acid isomer II	[31,32]
3.43	289.0722	289.0718	1.38	15 eV: 245.0911, 221.0974, 203.0787, 179.0379, 151.0459, 125.0292, 109.0336	C_15_H_14_O_6_	Epicatechin	[35,36]
3.45	337.0929	337.0929	0.00	15 eV: 191.0632, 173.0520, 163.0452	C_16_H_18_O_8_	Coumaroylquinic acid isomer II	[34]
4.21	193.0510	193.0506	2.07	20 eV: 178.0284, 134.0371	C_10_H_10_O_4_	Ferulic acid	[30]
4.67	431.1930 ^a^	431.1923 ^a^	1.62	10 eV: 205.1329, 179.0610, 161.0515, 113, 101.0273	C_20_H_32_O_10_	Vomifoliol glucoside	[37]
4.81	509.2240 ^a^	509.2240 ^a^	0.00	20 eV: 463.2207, 331.1887, 149.0478, 101.0225, 89.0247	C_21_H_36_O_11_	Linalool oxide apiosyl-glucoside	[34]
5.29	609.1463	609.1461	0.33	30 eV: 301,0363 300.0289, 271.0277, 178.9979	C_27_H_30_O_16_	Rutin	[30,38]
5.64	593.1513	593.1512	0.17	30 eV: 327.0588, 300.0277, 285.0414, 284.0341, 227.0353, 179.0078	C_27_H_30_O_15_	Kaempferol-glucoside-rhamnoside	[30]
7.06	493.2296	493.2291	1.01	10 eV: 447.2340	C_27_H_38_O_12_	Linalool or geraniol hexose-pentose	[37]
7.60	327.2169	327.2177	−2.44	20 eV: 291.1951, 229.1467, 211.1380, 171.1039, 85.0305	C_18_H_32_O_5_	Trihydroxy-octadecadienoic acid	[39]
8.03	329.2325	329.2323	0.61	15 eV: 311.2309, 229.1437, 211.1450, 171.1102	C_18_H_34_O_5_	Trihydroxy-octadecenoic acid	[39]

Note: ^a^ Formate adduct [M + HCOO]^−^.

## Data Availability

The raw data supporting the conclusions of this article will be made available by the authors upon request.

## Abbreviations

The following abbreviations are used in this manuscript:

AAPH	2,2′-Azobis(2-amidinopropane) dihydrochloride
AMPK	AMP-activated protein kinase
ANOVA	Analysis of variance
AUC	Area under the fluorescence decay curve
BCRJ	Rio de Janeiro Cell Bank
BSA	Bovine serum albumin
C	Control group
C1P	Ceramide-1-phosphate
carboxy-H_2_DCFDA	5-(and-6)-Carboxy-2′,7′-dichlorodihydrofluorescein diacetate
CAT	Catalase
CDNB	1-Chloro-2,4-dinitrobenzene
CEF	Compound Exchange Format
CERK	Ceramide kinase
DMEM	Dulbecco’s modified Eagle’s medium
DMSO	Dimethyl sulfoxide
DTNB	Dimethyl sulfoxide
EDTA	Ethylenediaminetetraacetic acid
EH	Aqueous extract of *Hancornia speciosa* leaves
EH50	Fatty acid-exposed cells treated with EH at 50 µg/mL
EH100	Fatty acid-exposed cells treated with EH at 100 µg/mL
ESI	Electrospray ionization
FA	Fatty acid-treated group
FBS	Fetal bovine serum
GAE	Gallic acid equivalents
GCL	Glutamate–cysteine ligase
GSH	Glutathione
GSSG	Oxidized glutathione
GST	Glutathione S-transferase
HepG2	Human hepatoblastoma-derived cell line
HMDB	Human Metabolome Database
HPLC	High-performance liquid chromatography
HPLC-ESI-MS/MS	High-performance liquid chromatography coupled with electrospray ionization tandem mass spectrometry
HPLC-ESI-QTOF-MS	High-performance liquid chromatography coupled to electrospray ionization quadrupole time-of-flight mass spectrometry
HPLC-MS	High-performance liquid chromatography–mass spectrometry
KEAP1	Kelch-like ECH-associated protein 1
KPE	Potassium phosphate buffer
LDH	Lactate dehydrogenase
MASLD	Metabolic dysfunction-associated steatotic liver disease
MPP	Mass Profiler Professional
MSI	Metabolomics Standards Initiative
MS/MS	Tandem mass spectrometry
MTT	3-(4,5-Dimethylthiazol-2-yl)-2,5-diphenyltetrazolium bromide
NADPH	Reduced nicotinamide adenine dinucleotide phosphate
NRF2	Nuclear factor erythroid 2-related factor 2
ORAC	Oxygen radical absorbance capacity
ORO	Oil Red O
PBS	Phosphate-buffered saline
QE	Quercetin equivalents
Q-TOF	Quadrupole time-of-flight
ROS	Reactive oxygen species
SIRT1	Sirtuin 1
SOD	Superoxide dismutase
SRB	Sulforhodamine B
SREBP-1c	Sterol regulatory element-binding protein 1c
TBHP	tert-Butyl hydroperoxide
TE	Trolox equivalents
TCA	Trichloroacetic acid
UV	Ultraviolet

## References

[1] Younossi Z.M., Kalligeros M., Henry L. (2025). Epidemiology of metabolic dysfunction-associated steatotic liver disease. Clin. Mol. Hepatol..

[2] Ipsen D.H., Lykkesfeldt J., Tveden-Nyborg P. (2018). Molecular mechanisms of hepatic lipid accumulation in non-alcoholic fatty liver disease. Cell. Mol. Life Sci..

[3] Chaurasia B., Summers S.A. (2015). Ceramides—Lipotoxic Inducers of Metabolic Disorders. Trends Endocrinol. Metab..

[4] Sutti S., Albano E. (2022). Oxidative stress in nonalcoholic fatty liver disease: A reappraisal of the role in supporting inflammatory mechanisms. Redox Exp. Med..

[5] García-Ruiz C., Fernández-Checa J.C. (2018). Mitochondrial Oxidative Stress and Antioxidants Balance in Fatty Liver Disease. Hepatol. Commun..

[6] Kupčová V., Fedelešová M., Bulas J., Kozmonová P., Turecký L. (2019). Overview of the Pathogenesis, Genetic, and Non-Invasive Clinical, Biochemical, and Scoring Methods in the Assessment of NAFLD. Int. J. Environ. Res. Public Health.

[7] Masenga S.K., Kabwe L.S., Chakulya M., Kirabo A. (2023). Mechanisms of Oxidative Stress in Metabolic Syndrome. Int. J. Mol. Sci..

[8] Arzumanian V.A., Kiseleva O.I., Poverennaya E.V. (2021). The Curious Case of the HepG2 Cell Line: 40 Years of Expertise. Int. J. Mol. Sci..

[9] Soret P.A., Magusto J., Housset C., Gautheron J. (2021). In vitro and in vivo models of non-alcoholic fatty liver disease: A critical appraisal. J. Clin. Med..

[10] Eynaudi A., Díaz-Castro F., Bórquez J.C., Bravo-Sagua R., Parra V., Troncoso R. (2021). Differential Effects of Oleic and Palmitic Acids on Lipid Droplet-Mitochondria Interaction in the Hepatic Cell Line HepG2. Front. Nutr..

[11] Arruda V.M., Azevedo G.T., Granato M.J.M.G., Matos A.C.P., Araújo T.G., Guerra J.F.D.C. (2024). Oxidative Stress and Annexin A2 Differential Expression in Free Fatty Acids-Induced Non-Alcoholic Fatty Liver Disease in HepG2 Cells. Int. J. Mol. Sci..

[12] Faria B.Q., Calixto P.S., Picheth G., Ferreira L.M., Rego F.G.M., Guerra J.F.C., Sari M.H.M. (2025). Palmitate-induced hepatic insulin resistance as an in vitro model for natural and synthetic drug screening: A scoping review of therapeutic candidates and mechanisms. Chem. Biol. Interact..

[13] Longhitano L., Distefano A., Amorini A.M., Orlando L., Giallongo S., Tibullo D., Lazzarino G., Nicolosi A., Alanazi A.M., Saoca C. (2023). (+)-Lipoic Acid Reduces Lipotoxicity and Regulates Mitochondrial Homeostasis and Energy Balance in an In Vitro Model of Liver Steatosis. Int. J. Mol. Sci..

[14] Simón J., Casado-Andrés M., Goikoetxea-Usandizaga N., Serrano-Maciá M., Martínez-Chantar M.L. (2020). Nutraceutical Properties of Polyphenols against Liver Diseases. Nutrients.

[15] Salomone F., Godos J., Zelber-Sagi S. (2016). Natural antioxidants for non-alcoholic fatty liver disease: Molecular targets and clinical perspectives. Liver Int..

[16] Fang X., Song J., Zhou K., Zi X., Sun B., Bao H., Li L. (2023). Molecular Mechanism Pathways of Natural Compounds for the Treatment of Non-Alcoholic Fatty Liver Disease. Molecules.

[17] Nakadate K., Ito N., Kawakami K., Yamazaki N. (2025). Anti-Inflammatory Actions of Plant-Derived Compounds and Prevention of Chronic Diseases: From Molecular Mechanisms to Applications. Int. J. Mol. Sci..

[18] Manach C., Scalbert A., Morand C., Rémésy C., Jiménez L. (2004). Polyphenols: Food sources and bioavailability. Am. J. Clin. Nutr..

[19] Vauzour D., Rodriguez-Mateos A., Corona G., Oruna-Concha M.J., Spencer J.P.E. (2010). Polyphenols and Human Health: Prevention of Disease and Mechanisms of Action. Nutrients.

[20] Polimeni L., Del Ben M., Baratta F., Perri L., Albanese F., Pastori D., Violi F., Angelico F. (2015). Oxidative stress: New insights on the association of non-alcoholic fatty liver disease and atherosclerosis. World J. Hepatol..

[21] Fraga C.G., Croft K.D., Kennedy D.O., Tomás-Barberán F.A. (2019). The effects of polyphenols and other bioactives on human health. Food Funct..

[22] Wang S., Du Q., Meng X., Zhang Y. (2022). Natural polyphenols: A potential prevention and treatment strategy for metabolic syndrome. Food Funct..

[23] Strassburg B.B.N., Brooks T., Feltran-Barbieri R., Iribarrem A., Crouzeilles R., Loyola R., Latawiec A.E., Oliveira Filho F.J.B., Scaramuzza C.A.D.M., Scarano F.R. (2017). Moment of truth for the Cerrado hotspot. Nat. Ecol. Evol..

[24] Cordeiro G.P., Araújo K.P., Rial R.C. (2026). Bioactive compounds associated with medicinal infusions and phytochemical preparations from Brazilian Cerrado plants: Phytochemical diversity and structural elucidation. Phytochem. Rev..

[25] Endringer D.C., Pezzuto J.M., Braga F.C. (2009). NF-κB inhibitory activity of cyclitols isolated from *Hancornia speciosa*. Phytomedicine.

[26] Reis V.H.D.O.T., Rodrigues B.M., Loubet Filho P.S., Cazarin C.B.B., Rafacho B.P.M., dos Santos E.E.F. (2022). Biotechnological potential of *Hancornia speciosa* whole tree: A narrative review from composition to health applicability. Heliyon.

[27] Santos U.P., Campos J.F., Torquato H.F.V., Paredes-Gamero E.J., Carollo C.A., Estevinho L.M., de Picoli Souza K., dos Santos E.L. (2016). Antioxidant, Antimicrobial and Cytotoxic Properties as Well as the Phenolic Content of the Extract from *Hancornia speciosa* Gomes. PLoS ONE.

[28] Leite S.P., Krause L.C., Jain S., Bjerk T.R. (2025). Scientific and Technical Insights into *Hancornia speciosa* Gomes for Biotechnological Applications. Compounds.

[29] Silva B.d.L.d.A., Vasconcelos M.A.d.S., Batista K.S., Lima M.d.S., Batista F.R.d.C., Cavalcante H.C., Toscano L.d.L.T., Silva A.S., D’Oliveira A.B., Alves A.F. (2024). Hepatoprotective, Lipid-Lowering and Antioxidant Effects of Mangaba Powder (*Hancornia speciosa*) Administered to Rats Fed a High-Fat Diet. Foods.

[30] Spínola V., Pinto J., Castilho P.C. (2015). Identification and quantification of phenolic compounds of selected fruits from Madeira Island by HPLC-DAD-ESI-MSn and screening for their antioxidant activity. Food Chem..

[31] Costa A.R., Almeida-Bezerra J.W., Gonçalves da Silva T., Pereira P.S., Borba E.F.O., Braga A.L., Fonseca V.J.A., Menezes S.A., Silva F.S.H., Fernandes P.A.S. (2021). Phytochemical profile and anti-Candida and cytotoxic potential of *Anacardium occidentale* L. (cashew tree). Biocatal. Agric. Biotechnol..

[32] Rabelo A.C.S., Araújo G.R.D., Lúcio K.D.P., Araújo C.M., Miranda P.H.D.A., Silva B.D.M., Carneiro A.C.A., Ribeiro É.M.D.C., Lima W.G.D., Souza G.H.B.D. (2017). Aqueous extract of *Baccharis trimera* improves redox status and decreases the severity of alcoholic hepatotoxicity. Rev. Bras. Farmacogn..

[33] Sobeh M., Mahmoud M.F., Abdelfattah M.A.O., Cheng H., El-Shazly A.M., Wink M. (2018). A proanthocyanidin-rich extract from *Cassia abbreviata* exhibits antioxidant and hepatoprotective activities in vivo. J. Ethnopharmacol..

[34] Li Q., Jin Y., Jiang R., Xu Y., Zhang Y., Luo Y., Huang J., Wang K., Liu Z. (2021). Dynamic changes in the metabolite profile and taste characteristics of Fu brick tea during the manufacturing process. Food Chem..

[35] Dou J., Lee V.S.Y., Tzen J.T.C., Lee M.R. (2007). Identification and comparison of phenolic compounds in the preparation of oolong tea manufactured by semifermentation and drying processes. J. Agric. Food Chem..

[36] Mbakidi-Ngouaby H., Pinault E., Gloaguen V., Costa G., Sol V., Millot M., Mambu L. (2018). Profiling and seasonal variation of chemical constituents from *Pseudotsuga menziesii* wood. Ind. Crops Prod..

[37] De Rosso M., Lonzarich V., Navarini L., Flamini R. (2022). Identification of new glycosidic terpenols and norisoprenoids (aroma precursors) in *C. arabica* L. green coffee by using a high-resolution mass spectrometry database developed in grape metabolomics. Curr. Res. Food Sci..

[38] Carneiro Junior W.O., Guimarães M.L.R., Freitas K.M., Pereira R.S., Pádua R.M., Campana P.R.V., Braga F.C. (2024). Structural characterization of a proanthocyanidin-rich fraction from *Hancornia speciosa* leaves and its effect on the release of pro-inflammatory cytokines and oxidative stress in THP-1 cells. J. Ethnopharmacol..

[39] Sakna S.T., Mocan A., Sultani H.N., El-Fiky N.M., Wessjohann L.A., Farag M.A. (2019). Metabolites profiling of *Ziziphus* leaf taxa via UHPLC/PDA/ESI-MS in relation to their biological activities. Food Chem..

[40] Bastos K.X., Dias C.N., Nascimento Y.M., da Silva M.S., Langassner S.M.Z., Wessjohann L.A., Tavares J.F. (2017). Identification of Phenolic Compounds from *Hancornia speciosa* (Apocynaceae) Leaves by UHPLC Orbitrap-HRMS. Molecules.

[41] de Araújo F.F., de Paulo Farias D., Neri-Numa I.A., Pastore G.M. (2021). Polyphenols and their applications: An approach in food chemistry and innovation potential. Food Chem..

[42] Pandey K.B., Rizvi S.I. (2009). Plant Polyphenols as Dietary Antioxidants in Human Health and Disease. Oxid. Med. Cell. Longev..

[43] Ivanova L., Uhlig S. (2008). A bioassay for the simultaneous measurement of metabolic activity, membrane integrity, and lysosomal activity in cell cultures. Anal. Biochem..

[44] Orellana E.A., Kasinski A.L. (2016). Sulforhodamine B (SRB) Assay in Cell Culture to Investigate Cell Proliferation. Bio Protoc..

[45] Kumar P., Nagarajan A., Uchil P.D. (2018). Analysis of Cell Viability by the Lactate Dehydrogenase Assay. Cold Spring Harb. Protoc..

[46] Yao H.R., Liu J., Plumeri D., Cao Y.B., He T., Lin L., Li Y., Jiang Y.Y., Li J., Shang J. (2011). Lipotoxicity in HepG2 cells triggered by free fatty acids. Am. J. Transl. Res..

[47] Li H.Y., Gan R.Y., Shang A., Mao Q.Q., Sun Q.C., Wu D.T., Geng F., He X.Q., Li H.B. (2021). Plant-based foods and their bioactive compounds on fatty liver disease: Effects, mechanisms, and clinical application. Oxid. Med. Cell. Longev..

[48] Luo L., Fang K., Dan X., Gu M. (2019). Crocin ameliorates hepatic steatosis through activation of AMPK signaling in db/db mice. Lipids Health Dis..

[49] Fang C., Pan J., Qu N., Lei Y., Han J., Zhang J., Han D. (2022). The AMPK pathway in fatty liver disease. Front. Physiol..

[50] Yao Y., Li L., Wang H., Yang Y., Ma H. (2022). Activated AMP-activated protein kinase prevents hepatic steatosis, oxidative stress and inflammation in primary chicken hepatocytes. Front. Physiol..

[51] Bae U.J., Park J., Park I.W., Chae B.M., Oh M.R., Jung S.J., Ryu G.S., Chae S.W., Park B.H. (2018). Epigallocatechin-3-gallate-rich green tea extract ameliorates fatty liver and weight gain in mice fed a high-fat diet by activating the sirtuin 1 and AMP-activated protein kinase pathway. Am. J. Chin. Med..

[52] Tilg H., Adolph T.E., Dudek M., Knolle P. (2021). Non-alcoholic fatty liver disease: The interplay between metabolism, microbes and immunity. Nat. Metab..

[53] Birben E., Sahiner U.M., Sackesen C., Erzurum S., Kalayci O. (2012). Oxidative Stress and Antioxidant Defense. World Allergy Organ. J..

[54] Halliwell B., Gutteridge J.M.C. (2015). Free Radicals in Biology and Medicine.

[55] Forman H.J., Zhang H., Rinna A. (2009). Glutathione: Overview of its protective roles, measurement, and biosynthesis. Mol. Asp. Med..

[56] Lu S.C. (2013). Glutathione synthesis. Biochim. Biophys. Acta Gen. Subj..

[57] Xu D., Xu M., Jeong S., Qian Y., Wu H., Xia Q., Kong X. (2019). The Role of Nrf2 in Liver Disease: Novel Molecular Mechanisms and Therapeutic Approaches. Front. Pharmacol..

[58] Zhou J., Zheng Q., Chen Z. (2022). The Nrf2 Pathway in Liver Diseases. Front. Cell Dev. Biol..

[59] Liu Z., Xia Y., Li B., Xu H., Wang C., Liu Y., Li Y., Li C., Gao N., Li L. (2014). Induction of ER stress-mediated apoptosis by ceramide via disruption of ER Ca^2+^ homeostasis in human adenoid cystic carcinoma cells. Cell Biosci..

[60] Yabu T., Shiba H., Shibasaki Y., Nakanishi T., Imamura S., Touhata K., Yamashita M. (2015). Stress-induced ceramide generation and apoptosis via the phosphorylation and activation of nSMase1 by JNK signaling. Cell Death Differ..

[61] Sztolsztener K., Chabowski A. (2024). Hepatic-metabolic activity of α-lipoic acid—Its influence on sphingolipid metabolism and PI3K/Akt/mTOR pathway in a rat model of metabolic dysfunction-associated steatotic liver disease. Nutrients.

[62] Gomez-Larrauri A., Ouro A., Trueba M., Gomez-Muñoz A. (2021). Regulation of cell growth, survival and migration by ceramide 1-phosphate—Implications in lung cancer progression and inflammation. Cell. Signal..

[63] Dong W., Li Q., Lu X., Lan J., Qiu Z., Wang X., Wang J., Zheng X., Chen S., Zhang C. (2024). Ceramide kinase-mediated C1P metabolism attenuates acute liver injury by inhibiting the interaction between KEAP1 and NRF2. Exp. Mol. Med..

[64] Jodynis-Liebert J., Kujawska M. (2020). Biphasic Dose-Response Induced by Phytochemicals: Experimental Evidence. J. Clin. Med..

[65] Chen X., Li H., Zhang B., Deng Z. (2022). The synergistic and antagonistic antioxidant interactions of dietary phytochemical combinations. Crit. Rev. Food Sci. Nutr..

[66] Huang D., Ou B., Hampsch-Woodill M., Flanagan J.A., Prior R.L. (2002). High-throughput assay of oxygen radical absorbance capacity (ORAC) using a multichannel liquid handling system coupled with a microplate fluorescence reader in 96-well format. J. Agric. Food Chem..

[67] Georgé S., Brat P., Alter P., Amiot M.J. (2005). Rapid determination of polyphenols and vitamin C in plant-derived products. J. Agric. Food Chem..

[68] Hossain M.A., Rahman S.M.M. (2011). Total phenolics, flavonoids and antioxidant activity of tropical fruit pineapple. Food Res. Int..

[69] Rahman I., Kode A., Biswas S.K. (2006). Assay for quantitative determination of glutathione and glutathione disulfide levels using enzymatic recycling method. Nat. Protoc..

[70] Habig W.H., Pabst M.J., Jakoby W.B. (1974). Glutathione S-transferases: The first enzymatic step in mercapturic acid formation. J. Biol. Chem..

[71] Madesh M., Balasubramanian K.A. (1998). Microtiter plate assay for superoxide dismutase using MTT reduction by superoxide. Indian J. Biochem. Biophys..

[72] Grilo L.F., Martins J.D., Cavallaro C.H., Nathanielsz P.W., Oliveira P.J., Pereira S.P. (2020). Development of a 96-well based assay for kinetic determination of catalase enzymatic-activity in biological samples. Toxicol. Vitr..

[73] Bradford M.M. (1976). A rapid and sensitive method for the quantitation of microgram quantities of protein utilizing the principle of protein-dye binding. Anal. Biochem..

[74] Sumner L.W., Amberg A., Barrett D., Beale M.H., Beger R., Daykin C.A., Fan T.W., Fiehn O., Goodacre R., Griffin J.L. (2007). Proposed minimum reporting standards for chemical analysis: Chemical Analysis Working Group (CAWG) Metabolomics Standards Initiative (MSI). Metabolomics.

